# Neurologic toxicity associated with immune checkpoint inhibitors: a pharmacovigilance study

**DOI:** 10.1186/s40425-019-0617-x

**Published:** 2019-05-22

**Authors:** Douglas B. Johnson, Ali Manouchehri, Alexandra M. Haugh, Henry T. Quach, Justin M. Balko, Benedicte Lebrun-Vignes, Andrew Mammen, Javid J. Moslehi, Joe-Elie Salem

**Affiliations:** 1 Department of Medicine, Vanderbilt University Medical Center, 777 PRB, 2220 Pierce Ave, Nashville, TN 37232 USA; 2 Department of Pharmacology, Sorbonne Université, INSERM CIC Paris-Est, AP-HP, ICAN, Regional Pharmacovigilance Centre, Pitié-Salpêtrière Hospital, Paris, France; 3 Muscle Disease Unit, Laboratory of Muscle Stem Cells and Gene Regulation, National Institute of Arthritis and Musculoskeletal and Skin Diseases, National Institutes of Health, Bethesda, MD USA

**Keywords:** Neurotoxicity, PD-1, CTLA-4, PD-L1, Neuropathy, Encephalitis, Myasthenia gravis, Guillain-Barre syndrome

## Abstract

**Background:**

Immune checkpoint inhibitors (ICI) produce durable antitumor responses but provoke autoimmune toxicities, including uncommon but potentially devastating neurologic toxicities. The clinical features, including the spectrum, timing, and outcomes, of ICI-induced neurologic toxicities are not well characterized.

**Methods:**

We performed disproportionality analysis using Vigibase, the World Health Organization pharmacovigilance database, comparing neurologic adverse event (AE) reporting in patients receiving ICIs vs. the full database. Neurologic AEs were classified by group queries using Medical Dictionary for Regulatory Activities, between database inception to September 28, 2018. Associations between ICIs and neurologic AEs were assessed using reporting odds ratios (ROR) and information component (IC). IC compares observed and expected values to find associations between drugs and AEs using disproportionate Bayesian reporting; IC_025_ (lower end of the IC 95% credibility interval) > 0 is considered statistically significant.

**Results:**

Among the full database, 18,518,994 AEs were reported, including 48,653 with ICIs. ICIs were associated with higher incidence of myasthenia gravis (0.47% of ICI reports vs. 0.04% of the full database, ROR 16.5 [95% CI 14.5–18.9]; IC_025_ 3.31), encephalitis (0.51% vs. 0.05%, ROR 10.4 [95% CI 9.2–11.8]; IC_025_ 3.15), peripheral neuropathy (1.16% vs. 0.67%, IC_025_ 0.68), and meningitis (0.15% vs. 0.06%, ROR 3.1 [95% CI 2.5–3.9]; IC_025_ 1.01). Myasthenia gravis and encephalitis were associated with anti-PD-1 whereas other neurologic AEs were associated with anti-CTLA-4. Myasthenia gravis was characterized by high fatality rates (~ 20%), early onset (median 29 days), and frequent concurrent myocarditis and myositis; whereas other neurologic AEs had lower fatality rates (6–12%), later onset (median 61–80 days), and were non-overlapping.

**Conclusions:**

ICIs produce a spectrum of distinct classes of neurologic AEs that can cause significant morbidity and mortality and tend to occur early and with class-specific associations.

**Electronic supplementary material:**

The online version of this article (10.1186/s40425-019-0617-x) contains supplementary material, which is available to authorized users.

## Introduction

Immune checkpoint inhibitors (ICI) have transformed the treatment landscape of numerous cancers, producing durable responses in a substantial fraction of patients [1]. Approved agents target programmed death-1 receptor (PD-1, nivolumab, pembrolizumab, cemiplimab), its ligand (PD-L1; atezolizumab, avelumab, durvalumab), and cytotoxic T lymphocyte antigen-4 (CTLA-4; ipilimumab) [2]. Toxic effects from these agents are related to removing nodes of self-tolerance and unleashing autoimmune-like phenomenon [3, 4]. Although usually manageable with corticosteroid administration, clinically severe events leading to morbidity and even mortality may complicate treatment [5].

Neurologic toxicities have emerged as clinically relevant complications of ICI. Case series have reported limited numbers of autoimmune-like, inflammatory events including encephalitis, aseptic meningitis, myasthenia gravis, and Guillain-Barre Syndrome which may occur, in aggregate, in 1–5% of treated patients [6–11]. However, a systematic analysis of the timing, spectrum, clinical associations, and outcomes of these uncommon events has not been performed in a large number of patients. Further, it remains unclear whether ICI are associated with other, more common neurologic events (such as cerebrovascular accident, seizures, multiple sclerosis, dementia, etc.). Defining the severe toxicities of ICI remains a critical objective given the rapidly increasing use of these agents, and long-term survival experienced by responding patients. While pharmacovigilance data may lack detailed clinical information, using this approach may help rigorously identify drug-toxicity associations. Herein, we leverage a large pharmacovigilance database (Vigibase), which has been used to characterize other ICI-induced toxicities [5, 12–16], to further define the neurologic toxicities associated with ICI.

## Methods

### Study design and data sources

This observational, retrospective, pharmacovigilance study is a disproportionality analysis based on adverse drug reactions reported in VigiBase, the WHO database of global, deidentified individual case safety reports (ICSRs), which includes reports from more than 130 countries [17]. VigiBase is managed by the Uppsala Monitoring Centre (UMC; Uppsala, Sweden), and contains more than 18.5 million ICSRs submitted by national pharmacovigilance centers since 1967. These reports originate from healthcare professionals, patients, or pharmaceutical companies, and are generally notified post-marketing.

### Procedures

This study included all neurologic toxicities classified by group queries according to the Medical Dictionary for Regulatory Activities (MedDRA; Additional file 1: Table S1), between inception in November 14, 1967, and September 28, 2018. We categorized neurologic entities in the MedDRA classification based on the underlying pathophysiology, due to the overlap between different neurologic symptoms. Neurologic irAEs assessed in the analysis was limited to those suspected to be caused by ICIs. Each report in VigiBase contains administrative data (date of reporting, country of origin, qualification of reporter), patient characteristics (age and sex), drug characteristics (indication for treatment [cancer type], administration start and end dates, dose and regimen, route of administration), and reactions or events (reported terms including MedDRA classification terms, onset date, end date, seriousness, final outcome). We included ICIs in this study, including antibodies targeting PD-1 (nivolumab and pembrolizumab), PD-L1 (atezolizumab, avelumab, durvalumab), and CTLA-4 (ipilimumab, tremelimumab). Severe adverse events were defined as life-threatening events or those causing death, hospitalization, persistent or clinically significant disability, congenital anomaly, birth defect, or other significant medically important condition.

### Statistical analysis

VigiBase allows for case/non-case analysis (also known as disproportionality analysis), which we used to study if suspected drug-induced neurologic events were differentially reported with ICIs as compared to neurologic events reported in the entire database with all the suspected drug-induced adverse reactions. Disproportionality analysis was also used to assess neurologic toxicities with different ICI regimens: anti-CTLA-4 monotherapy versus anti-PD-1/PD-L1 monotherapy versus combination ICI therapy (anti-CTLA-4 and anti-PD-1/PD-L1 combination therapy). Disproportionality analysis compares the proportion of selected specific adverse-drug-reaction reported for a single or a group of drugs (e.g. ICI) with the proportion of the same adverse-drug-reaction for a control group of drugs (e.g. full database). The denominator in these analyses is the overall adverse-drug-reactions reported for each group of drugs. If the proportion of an adverse-drug-reaction is greater in patients exposed to a group of drug (cases) than in patients not exposed to this drug (non-cases), this suggests an association between the specific drug and the reaction and is a potential signal for safety. Disproportionality can be either calculated by the information component (IC) or reporting odds-ratio (ROR) when using full database as comparator, and only ROR when using different drug regimen subgroups as comparators.

Calculation of the IC, using a Bayesian confidence propagation neural network, was specifically developed and validated by UMC as a flexible, automated indicator value for disproportionate reporting that compares observed and expected drug-AE associations to find new drug-AE signals with identification of probability difference from the background data (full database) [18]. Probabilistic reasoning in intelligent systems (information theory) has been proven effective to manage large data sets, is robust in handling incomplete data, and may be used with complex variables. Information theory tools are ideal for finding drug-AE combinations with other variables, which are highly associated compared to the generality of the stored data [18]. Several examples with IC have been first validated showing the power of the technique to find signals very early after drug approval (e.g. captopril ± coughing) and to avoid false positives where a common drug and a common AE association occur in the database, only because the drug is widely used and the AE frequently reported (e.g. digoxin ± acne; digoxin ± rash) [18, 19]. Using VigiBase, this approach has recently been proven effective to characterize the spectrum of cardiovascular immune related adverse events associated with ICIs [16].

The statistical formula is as follows to calculate IC,

1 $$ \mathrm{IC}=\log 2\left(\left({\mathrm{N}}_{\mathrm{observed}}+0.5\right)/\left({\mathrm{N}}_{\mathrm{expected}}+0.5\right)\right) $$

2 $$ \mathrm{where}\ {\mathrm{N}}_{\mathrm{expected}}=\left({\mathrm{N}}_{\mathrm{drug}}\ast {\mathrm{N}}_{\mathrm{effect}}\right)/{\mathrm{N}}_{\mathrm{total}} $$

N_expected_: the number of case reports expected for the drug-adverse effect combination.

N_observed_: the actual number of case reports for the drug- adverse effect combination.

N_drug_: the number of case reports for the drug, regardless of adverse effects.

N_effect_: the number of case reports for the adverse effect, regardless of drug.

N_total_: the total number of case reports in the database.

IC_025_ is the lower end of a 95% credibility interval for the Information Component. A positive IC_025_ value (> 0) is the traditional threshold used in statistical signal detection at UMC [18, 19]. IC_025_ have only been validated for comparison of a drug versus full database and cannot be used to compare disproportionate reporting within different ICI regimens.

Disproportionality for neurologic irAE reporting as a function of variable ICI regimen was estimated by calculating the ROR Chi-square (Graphpad Prism 7), described elsewhere and detailed in Additional file 1: Table S2 [20–22]. The lower end of ROR 95% confidence interval (CI) ≥1 is the threshold used for significant statistical signal detection. Characteristics of cases were described in terms of means (± standard deviation) or medians (with interquartile range) for quantitative variables, and in terms of effective and proportion for qualitative ones. Time to toxicity was compared between different toxicities using the logrank test. Categorical variables were compared between toxicities using chi-square testing.

## Results

A total of 48,653 adverse events were reported with ICI drugs, from a total number of 18,518,994 ICSRs reported in the full VigiBase dataset. Since ICI reports began in 2008, we also note that 14,627,365 ICSRs have been reported in VigiBase from 2008 – September 28, 2018. The numbers of different neurologic toxicities associated with ICI and in the full database are shown in Tables 1 and 2.

**Table 1:** Neurologic immune-related adverse events reported with ICIs versus those reported in the full database from VigiBase, from Nov 14, 1967, to September 28, 2018

	Overall ICIs	Full database (starting 1967)	IC / IC_025_
Total number of ICSRs available	48,653	18,518,994	
Number of ICSRs by irAE subgroups			
Neuromuscular junction dysfunction (myasthenia gravis)	**228 (0.47%)**	**7455 (0.04%)**	**3.51/3.31**
Noninfectious encephalitis and/or myelitis	**250 (0.51%)**	**9267 (0.05%)**	**3.33/3.15**
Cerebral artery vasculitis	**34 (0.07%)**	**1206 (0.01%)**	**3.23/2.71**
Peripheral neuropathy	**564 (1.16%)**	**123,463 (0.67%)**	**0.80/0.68**
- Guillain-Barre syndrome	**122 (0.25%)**	**9508 (0.05%)**	**2.27/2.00**
- Chronic polyneuropathies	23 (0.05%)	6428 (0.03%)	0.43/−0.22
- Mononeuropathies	42 (0.09%)	17,075 (0.09%)	−0.09/− 0.58
Noninfectious meningitis	**72 (0.15%)**	**10,532 (0.06%)**	**1.36/1.01**
Hemorrhagic central nervous system vascular conditions	386 (0.79%)	195,577 (1.06%)	−0.41/− 0.56
Cranial nerve disorders (excluding neoplasms)	226 (0.46%)	112,639 (0.61%)	−0.39/− 0.58
Cerebral ischemia	332 (0.68%)	174,768 (0.94%)	−0.47/− 0.63
Spinal cord and nerve root disorders	27 (0.06%)	11,875 (0.06%)	−0.21/− 0.80
Speech and language abnormalities	215 (0.44%)	125,871 (%)	−0.62/− 0.82
Seizures	291 (0.60%)	238,924 (0.68%)	−1.11/−1.28
Headaches	776 (1.59%)	731,460 (3.95%)	−1.31/− 1.41
Coma states	56 (0.12%)	43,228 (0.23%)	−1.01/− 1.41
Extrapyramidal syndrome	854 (1.76%)	840,831 (4.54%)	−1.37/− 1.47
Sensory abnormalities	520 (1.07%)	551,559 (2.98%)	−1.48/− 1.60
Dementia	21 (0.04%)	15,706 (0.08%)	−0.96/−1.64
Movement disorders	396 (0.81%)	427,006 (2.31%)	−1.50/− 1.65
Vertigos	89 (0.18%)	91,034 (0.49%)	−1.42/− 1.74
Nervous system neoplasms benign	5 (0.01%)	2302 (0.01%)	−0.25/−1.78
Sleep disturbances	239 (0.49%)	314,528 (1.70%)	−1.79/− 1.98
Psychosis and psychotic disorders	183 (0.38%)	284,048 (1.53%)	−2.03/−2.24
Demyelinating disorders	38 (0.08%)	87,190 (0.47%)	−2.58/−3.07

Data are *N* (%) unless otherwise stated. ICIs refers to any ICSRs reported for treatment with nivolumab, pembrolizumab, atezolizumab, avelumab, durvalumab, ipilimumab, or tremelimumab. A positive IC_025_ value (> 0) is the traditional threshold used in statistical signal detection with VigiBase. *ICSRs* individual case safety reports. *ICIs* immune checkpoint inhibitors. *IC* information component. IC025 = lower end of a 95% credibility interval for the IC

Bold text denotes statistically significant differences

**Table 2:** Selected neurological adverse events (detected as signals) reported for ICIs versus the full database from VigiBase, from Jan 1, 2008, to September 28, 2018

Total number of ICSRs	Overall ICIs (n:)			Full database (full; starting 2008^a^; *N*:14,627,365)	ROR and 95% CI [,] anti-PD-1 or anti-PD-L1 vs anti-CTLA-4 monotherapy	ROR and 95% CI [,] combination ICIs vs monotherapy	ROR and 95% CI [,] ICIs vs full database
	MONO (*N*:43,960)		COMB (*N*:4693)				
	MONO-PD1 (*N*:34,401)	MONO-CTLA4 (*N*:9559)					
Number of ICSRs by Neuro-ADR subgroup							
Neuromuscular junction dysfunction	197 (0.57%)	14 (0.15%)	17 (0.36%)	4380 (0.03%)	**3.9 [2.3–6.8]**	0.8 [0.5–1.2]	**16.5 [14.5–18.9]**
Non-infectious encephalitis and/or myelitis	186 (0.54%)	21 (0.22%)	43 (0.92%)	7460 (0.05%)	**2.5 [1.6–3.9]**	**2 [1.4–2.7]**	**10.4 [9.2–11.8]**
Guillain-Barre syndrome	64 (0.19%)	37 (0.39%)	21 (0.45%)	7962 (0.05%)	**0.48 [0.32–0.72]**	**2 [1.2–3.1]**	**4.7 [3.9–5.6]**
Non-infectious meningitis	36 (0.10%)	20 (0.21%)	16 (0.34%)	6986 (0.04%)	**0.5 [0.29–0.86]**	**2.7 [1.5–4.7]**	**3.1 [2.5–3.9]**

*Abbreviations*: *Mono* monotherapy, *COMB* combination therapy, *PD1* Programmed death-1/ligand-1, *CTLA4* cytotoxic T lymphocyte antigen-4

Data are *N* (%) unless otherwise stated. ICIs refers to any ICSR reported for treatment with nivolumab, pembrolizumab, atezolizumab, avelumab, durvalumab, ipilimumab, or tremelimumab. Anti-PD-1 or anti-PD-L1 monotherapy refers to any ICSR associated with any of the following five drugs only when used alone: nivolumab, pembrolizumab, atezolizumab, avelumab, or durvalumab. Anti-CTLA-4 monotherapy refers to any ICSR associated with ipilimumab or tremelimumab alone. Combination ICIs refers to any ICSR reported with at least one anti-PD-1 or anti-PD-L1 drug combined with an anti-CTLA-4 drug. ICSRs = individual case safety reports. ICIs = immune checkpoint inhibitors. ROR = reporting odds ratio

^a^First reports of ICSRs associated with ICIs started in 2008

Bold text denotes statistically significant differences

We identified five broad categories of neurologic events associated with ICI treatment compared with reporting from the full database. ICIs were associated with higher reporting of neuromuscular junction dysfunction (0.47% of reports with ICIs vs. 0.04% for the full database, ROR 16.5 [95% CI 14.5–18.9]; IC_025_ 3.31), non-infectious encephalitis and/or myelitis (0.51% vs. 0.05%, ROR 10.4 [95% CI 9.2–11.8]; IC_025_ 3.15), cerebral artery vasculitis (0.07% vs. 0.01%; ROR 10.6 [95% CI 7.5–14.9]; IC_025_ 2.71), peripheral neuropathy (1.16% vs. 0.67%, IC_025_ 0.68), and non-infectious meningitis (0.15% vs. 0.06%, ROR 3.1 [95% CI 2.5–3.9]; IC_025_ 1.01). The increased reporting of peripheral neuropathy was in part driven by acute polyneuropathies, specifically Guillain-Barre syndrome which comprised 22% (*n* = 122/564) of peripheral neuropathy cases and were disproportionately associated with ICI (0.25% vs. 0.05%, ROR 4.7 [95% CI 3.9–5.6], IC_025_ 2.00). Notably, most cases of cerebral artery vasculitis reported were temporal arteritis (*n* = 23, 67.7%), although cerebral vasculitis (*n* = 8) was also observed. Since we recently described temporal arteritis and other vasculitis syndromes associated with ICI, [16] we chose not to focus on these further; characteristics of patients with CNS vasculitis are listed in Additional file 1: Table S3. Other neurologic events, including hemorrhagic or ischemic strokes, seizures, headaches, extrapyramidal syndromes, dementia, sleep disturbances, psychotic disorders, or demyelinating disorders did not have increased reporting with ICIs (Table 1).

**Fig. 1:**
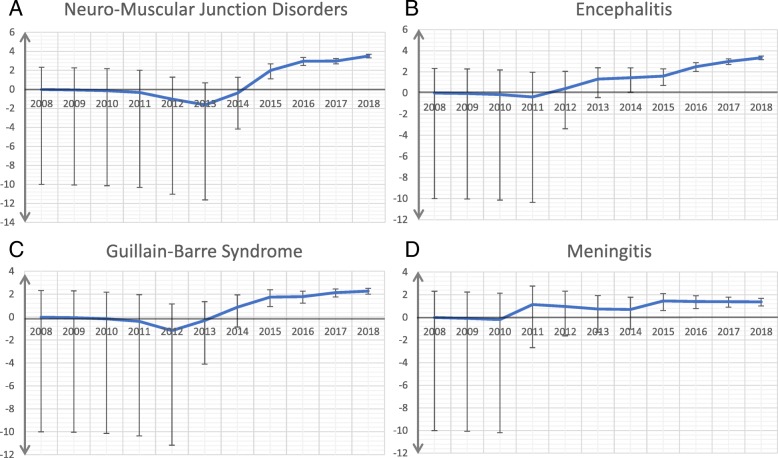
Information component and its 95% credibility interval over time for (**a**) neuromuscular junction disorders, (**b**) encephalitis/myelitis, (**c**) Guillain-Barre Syndrome, and (**d**) meningitis

The associations of neurologic irAEs with class of ICI (anti-PD-1/PD-L1 vs. anti-CTLA-4 vs. combination) are listed in Table 2. Notably, we observed distinct events associated with different classes. Neuromuscular junction dysfunction (myasthenia gravis) was over-reported in patients treated with anti-PD-1/PD-L1 compared with anti-CTLA-4 (ROR 3.9, 95% CI 2.3–6.8). Non-infectious encephalitis/myelitis was reported more with anti-PD-1/PD-L1 than with anti-CTLA-4 (ROR 2.5, 95% CI 1.6–3.9) and with combination therapy compared with monotherapy (ROR 2.0, 95% CI 1.4–2.7). By contrast, Guillain-Barre syndrome (GBS) (ROR 0.48, 95% CI 0.32–0.72) and non-infectious meningitis (ROR 0.5, 95% CI 0.29–0.86) were less frequently reported with anti-PD-1/PD-L1 compared with anti-CTLA4 monotherapy, and were more frequently associated with combination PD-1/PD-L1 + CTLA-4 blockade compared with monotherapy (ROR 2.0 [95% CI 1.2–3.1] and ROR 2.7 [95% CI 1.5–4.7] for GBS and meningitis, respectively). Similar OR were observed with individual anti-PD-1 vs. anti-PD-L1 drugs, although a low number of anti-PD-L1 associated events were noted (data not shown). IC values and their 95% credibility intervals over time are shown in Fig. 1 for neuromuscular junction disorders, encephalitis, GBS, and meningitis.

To assess the clinical features of these neurologic irAEs associated with ICIs, we described the clinical characteristics of patients who developed neuromuscular junction dysfunction (*n* = 228), noninfectious encephalitis/myelitis (*n* = 250), Guillain-Barre Syndrome (*n* = 122), and noninfectious meningitis (*n* = 72) (Table 3). Most cases were reported in 2017–2018 (61–78% of cases), reflecting the substantially increased use of ICIs in recent years. Toxicities were substantially more common in men than women, ranging from 53 to 65% men depending on the toxicity. Encephalitis/myelitis and meningitis appeared to occur in slightly younger patients (median ages 58.6 and 56.3 years, respectively) compared with myasthenia gravis and GBS (median 70.3 and 65 years, respectively). Cases largely were reported in patients with lung cancer (33% of all cases; *n* = 188/574) and melanoma (36%; *n* = 206/574). Comparing lung cancer and melanoma, myasthenia gravis and encephalitis/myelitis were more common in lung cancer (163 vs. 103 cases) whereas GBS and meningitis were more common in melanoma (103 vs. 25 cases; *p* < 0.0001) although this may reflect the differential use of causative therapies in these cancers (e.g. events associated with ipilimumab occurred more often in melanoma patients).

**Table 3:** : Clinical characteristics of patients with ICI induced neurotoxicities

Characteristics	Myasthenia gravis *N* (%)^a^	Encephalitis/ myelitis *N* (%)^a^	Guillain-Barre syndrome *N* (%)^a^	Non-infectious meningitis *N* (%)^a^
Total number	228	250	122	72
Reporting year				
2018 (through September)	101 (44.30)	107 (42.80)	45 (36.9)	20 (27.78)
2017	73 (32.02)	90 (36.00)	46 (37.7)	24 (33.33)
2016	39 (17.11)	38 (15.20)	17 (13.9)	13 (18.06)
2015	12 (5.26)	8 (3.20)	7 (5.7)	9 (12.50)
2014	3 (1.32)	3 (1.20)	6 (4.9)	3 (4.17)
2012–2013	0	4 (1.60)	1 (0.82)	3 (4.17)
Gender				
Male	125 (60.98)	140 (63.35)	71 (65.74)	35 (53.03)
Female	80 (39.02)	81 (36.94)	37 (34.26)	31 (46.97)
*Data available*	*205 (89.91)*	*221 (88.40)*	*108 (88.52)*	*66 (91.67)*
Age at onset, mean ± SD, years	70.28 ± 10.50	58.69 ± 15.98	65.01 ± 14.41	56.25 ± 14.63
[min-max]	[32–86]	[7–86]	(25–98)	[28, 86]
*Data available*	*122 (53.51)*	*162 (64.80)*	*76 (62.29)*	*51 (70.83)*
Drugs				
*Monotherapy with Anti PD-1/PD-L1*	197 (86.40)	186 (74.40)	64 (52.46)	36 (50.00)
*- Nivolumab*	68 (34.52)	127 (68.28)	33 (51.56)	23 (63.89)
*- Pembrolizumab*	116 (58.88)	36 (19.35)	27 (42.19)	9 (25.00)
*- Atezolizumab*	9 (4.57)	20 (10.58)	3 (4.69)	4 (11.11)
*- Durvalumab*	1 (0.51)	3 (1.59)	0 (0)	0 (0)
*- Avelumab*	3 (1.52)	0 (0)	1 (0.82)	0 (0)
*Monotherapy with Anti CTLA-4*	14 (6.14)	21 (8.40)	37 (30.33)	20 (27.78)
*- Ipilimumab*	14 (100)	20 (100)	37 (100)	20 (100)
*Combination therapy*	17 (7.46)	43 (17.20)	21 (17.21)	16 (22.22)
*- Nivolumab + Ipilimumab*	15 (88.24)	40 (93.02)	20 (95.24)	16 (100)
*- Pembrolizumab + Ipilimumab*	1 (5.88)	3 (6.98)	1 (4.76)	0 (0)
*- Tremelimumab + Durvalumab*	1 (5.88)	0 (0)	0 (0)	0 (0)
Indications				
Lung cancer	73 (39.67)	90 (38.96)	18 (18.75)	7 (11.11)
Malignant melanoma	47 (25.54)	56 (24.24)	65 (67.71)	38 (60.32)
Renal cell carcinoma	25 (13.59)	13 (5.62)	5 (5.21)	5 (7.94)
Other	39 (21.20)	51 (20.40)	8 (8.33)	13 (20.63)
*Data available*	*184 (80.70)*	*231 (85.20)*	*96 (78.69)*	*63 (87.50)*
Time to irAE onset, days:				
Median, [IQR]	29 [24–53]	61 [18–153]	65.5 [29–124]	68 [27–134]
[min-max]	[6–132]	[1–841]	[2–995]	[8–400]
*Data available*	*45 (19.74)*	*72 (28.80)*	*34 (27.87)*	*23 (31.51)*
Death	44 (19.30)	32 (12.80)	13 (10.66)	6 (8.33)
Concurrent neurologic symptoms/syndromes				
Myasthenia gravis	N/A	1 (0.40)	1 (0.82)	0 (0)
Encephalitis/myelitis	1 (0.44)	N/A	1 (0.82)	5 (6.94)
Cerebral vasculitis	0 (0)	1 (0.40)	0 (0)	1 (1.39)
Guillain Barre syndrome	1 (0.44)	1 (0.40)	N/A	2 (2.78)
Peripheral Neuropathy	0 (0)	4 (1.60)	N/A	0 (0)
Meningitis	0 (0)	5 (5.00)	2 (1.64)	N/A
Demyelination	0 (0)	5 (2.00)	0 (0)	0 (0)
Seizure	1 (0.44)	10 (4.00)	0 (0)	0 (0)
Stroke	0 (0)	2 (0.80)	0 (0)	4 (5.56)
Blindness (unilateral or bilateral)	1 (0.44)	0 (0)	0 (0)	0 (0)
Coma/loss of consciousness	1 (0.44)	4 (1.60)	0 (0)	0 (0)
Other irAEs				
Colitis/diarrhea	4 (1.75)	4 (1.60)	3 (2.46)	3 (4.17)
Pneumonitis	1 (0.44)	5 (2.00)	0 (0)	2 (2.78)
Myocarditis	20 (10.52)	3 (1.20)	0 (0)	1 (1.39)
Myositis	37 (16.23)	2 (0.80)	0 (0)	1 (1.39)
Dermatitis	2 (0.88)	12 (4.80)	3 (2.46)	3 (4.17)
Thyroiditis/hypothyroidism	7 (3.07)	6 (2.40)	5 (4.10)	1 (1.39)
Hypophysitis/hypopituitarism	0 (0)	5 (2.00)	1 (0.82)	5 (6.94)
Hepatitis	11 (4.82)	2 (0.80)	3 (2.46)	1 (1.39)
Nephritis	0 (0)	2 (0.80)	0 (0)	1 (1.39)
Other	3 (1.32)	10 (4.00)	2 (1.64)	7 (9.72)
None	154 (67.54)	205 (82.00)	110 (90.16)	46 (63.89)

^a^Data available = 100% unless noted

Abbreviations: *CTLA-4* cytotoxic T-lymphocyte-associated protein 4, *ICI* immune checkpoint inhibitor, *IQR* interquartile range, *irAE* immune related adverse event, *[min-max]* minimum-maximum, *PD-1* programmed cell death protein 1, *PD-L1* programmed cell death ligand 1, *SD* standard deviation, *N/A* not applicable

**Fig. 2:**
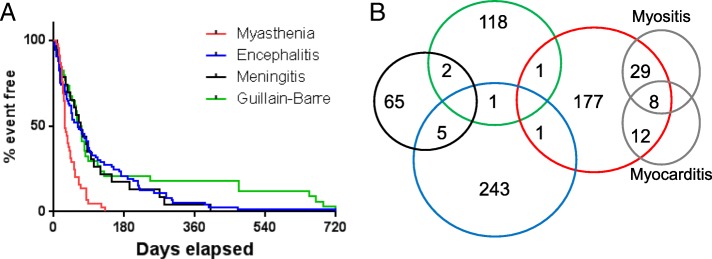
**a** Time to event for different categories of neurotoxicities. **b** Modified Venn diagram showing overlap between distinct classes of neurotoxicities (color scheme the same as in A), as well myositis and myocarditis. Blank cells have no cases for neurotoxicities (e.g. no reports of concurrent myasthenia gravis, GBS, and encephalitis) or were beyond the scope of this study (myositis and myocarditis cases without overlapping neurotoxicities)

Neurologic events generally occurred within the first three months after starting ICI therapy; however myasthenia gravis (median 29 days) had more rapid onset than other events (median onset 61–80 days; *p* < 0.001) (Fig. 2a). Myasthenia gravis also had the highest fatality rates (44/228, 19.3%) compared with other neurologic toxicities (51/444, 11.5%; *p* = 0.024). Myasthenia gravis presenting with both myocarditis and myositis had the highest death rate (5/8; 62.5%), compared with myasthenia gravis alone (29/179; 16.2%), or myasthenia gravis presenting with either myositis alone (6/29; 20.7%), or with myocarditis alone (4/12; 33%). Among patients who died with available data, median time to death was 43 days (myasthenia gravis), 64.5 days (Guillain-Barre), 60.8 days (encephalitis), and 42 days (meningitis). Neurologic AEs were rarely overlapping, with the exception of encephalitis and meningitis in 5 patients (Fig. 2b). Most patients did not have concurrent severe non-neurologic irAEs, with the exception of patients with myasthenia gravis, which often had myocarditis (8.8%), myositis (16.2%), or both. Myasthenia gravis (74/228; 32.5%) and meningitis (26/72; 36.1%) were more likely to have concurrent irAEs compared with other neurotoxicities (45/250, 18% and 12/122, 9.9% for encephalitis and GBS, respectively; *p* < 0.0001). Additional clinical descriptions are shown in Additional file 1: Tables S4-S7.

## Discussion

This study reports, to our knowledge, the largest characterization of neurologic irAEs associated with ICIs by detailed analysis of a global, WHO database (VigiBase). ICIs were significantly associated with over reporting of five distinct categories of neurologic toxicities: neuromuscular dysfunction (myasthenia gravis), encephalitis/myelitis, cerebral vasculitis, Guillain-Barre Syndrome, and non-infectious meningitis. While the clinical features of a limited number of these cases have been reported previously, this is the first effort to systematically associate their occurrence with ICI use, and characterize a large population of affected patients.

ICIs function by removing key regulators of immune tolerance; thus toxicities affecting other organ systems have largely been related to autoimmune-type and inflammatory phenomenon. The events observed in our study largely recapitulate these findings, with inflammatory manifestations (encephalitis, vasculitis, meningitis), and/or autoimmune syndromes (myasthenia gravis and GBS). Interestingly, these events were differentially associated either with single-agent anti-PD-1/PD-L1 (myasthenia gravis and encephalitis) or anti-CTLA-4 (GBS and meningitis), and were generally increased by combination ICI therapy. Neurotoxicities tended to occur fairly early on therapy (median onset within 3 months for all categories), although only myasthenia gravis had a hyperacute onset (similar to myocarditis or fatal irAEs) [5, 12]. The severity of these events varied, with fatality rates similar to irAEs involving other organ systems (approximately 10% for most events) [5]. Notably, the low number of events observed (concordant with lower use overall) of anti-PD-L1 drugs (vs. anti-PD-1) highlights the need to study differences between these classes.

Myasthenia gravis appeared to have the most fulminant presentation, with a nearly 20% fatality rate, early onset (median 29 days), and frequent association with myositis and myocarditis (16 and 10%, respectively). Myasthenia gravis may progress to involve the respiratory muscles, which may partially explain the high fatality rate. The association with myocarditis and myositis may also contribute [12, 16], and will require additional biologic characterization to explain this co-occurrence. Many cases of myasthenia gravis also reported cardiac arrhythmias or myocardial infarction even in cases where myocarditis was not reported, suggesting that myocarditis may be more common than recognized, and highlighting the need for diagnostic assessment for myocarditis and myositis (assessing creatinine kinase and troponin I) in patients with myasthenia gravis. Notably, the myasthenia gravis associated with ICI may be distinct from the de novo syndrome, as acetylcholinesterase receptor antibodies are frequently negative, and there is a high incidence of concurrent myositis [13].

Importantly, we did not observe additional signals of neurotoxicities. One somewhat surprising negative finding was the lack of association between ICI and demyelinating disorders such as multiple sclerosis. Multiple sclerosis is characterized by demyelination, axonal degeneration, and intense inflammation in affected lesions. This inflammation is characterized by T cell, B cell, and macrophage involvement, and improves with corticosteroids and interferon-β (and would thus be predicted to be triggered by ICI as with numerous other inflammatory disorders) [23]. Interestingly, demyelinating disorders actually appeared to be negatively correlated with ICI use (0.08% of ICI reports vs. 0.47% for the full database; IC_025_: − 3.07), although this study was not designed to conclusively exclude drug-AE associations. The lack of associations with other neurologic disorders was also an important finding, given evidence that suggests inflammation may play a critical role in diverse neurologic conditions such as dementia, cerebrovascular disease, amytrophic lateral sclerosis, and others [24–26]. However, long-term data will be needed to definitively rule out such associations.

There are several limitations to the study intrinsic to Vigibase. First, adverse event reporting is voluntary, and comes from heterogeneous sources (e.g. physicians, pharmacists, other clinicians), thus raising the possibility of incomplete information. However, > 130 countries contribute to the database, thus ensuring an unparalleled global assessment in diverse clinical settings. Second, detailed clinical information and diagnostic criteria is unavailable, thus limiting our assessment to the treating clinician’s reporting (e.g. we do not have electromyogram or cerebrospinal fluid data to confirm reported diagnoses). This may introduce bias in several directions, including either under-reporting (e.g. only reporting the most severe or obvious cases), or over-reporting (reporting cases without a firmly established diagnosis). This study does, however, complement other detailed descriptions of these clinical syndromes [6–11]. Third, we are unable to definitively determine the incidence of each event using Vigibase, although other studies have reported incidence of neurologic toxicities in the range of 1–5% [7, 8]. Fourth, changing awareness of toxicities over time could influence reporting. For example, our group and others published high-profile papers showing a link between myocarditis, myositis, and myasthenia gravis [12, 27]. These and other publications could have prompted improved awareness and potentially increased reporting over time. Finally, comparisons with anti-CTLA-4 and anti-PD-1/PD-L1 monotherapy could be confounded by disease-specific (rather than treatment specific) factors. Melanoma patients largely comprise the anti-CTLA-4 group, and may have distinct demographic and toxicity proclivities compared with the more pan-tumor population treated with anti-PD-1/PD-L1. As with other pharmacovigilance studies, this study allows for signal detection in a large population, which will need prospective and long-term validation of findings.

## Conclusions

In conclusion, several categories of neurologic toxicities were strongly associated with ICI use relating to CNS inflammation (encephalitis/myelitis, meningitis, and CNS vasculitis) or peripheral neuromuscular autoimmune disorders (Guillain-Barre and myasthenia gravis). Of equal importance, no signals of other neurologic toxicities were observed, including demyelinating disorders or cerebrovascular disease. Clinicians should be aware of, and monitor for these potentially severe irAEs in patients receiving ICI therapy.

## Additional file

Additional file 1: **Table S1.** Neuro-psychiatric adverse events grouping as a function of Medical Dictionary for Regulatory Activities (MedDRA) Classification Version 21.0. **Table S2.** Example of calculating reporting odds ratio (ROR) in Vigibase. **Table S3.** Clinical characteristics of patients with ICI-associated cerebral vasculitis collected from VigiBase. **Table S4.** Clinical characteristics of patients with ICI-associated myasthenia gravis collected from VigiBase. **Table S5.** Clinical characteristics of patients with ICI-associated encephalitis/myelitis collected from VigiBase. **Table S6.** Clinical characteristics of patients with ICI-associated Guillain-Barre Syndrome collected from VigiBase. **Table S7.** Clinical characteristics of patients with ICI-associated non-infectious meningitis collected from VigiBase. (DOCX 38 kb)
