# Supplementary material for: Neurologic toxicity associated with immune checkpoint inhibitors: a pharmacovigilance study
Source: J Immunother Cancer. 2019 May 22;7:134. doi: 10.1186/s40425-019-0617-x (PMC6530194; doi:10.1186/s40425-019-0617-x)
Supplement: Supplementary file 1 — Table S1. Neuro-psychiatric adverse events grouping as a function of Medical Dictionary for Regulatory Activities (MedDRA) Classification Version 21.0. Table S2. Example of calculating reporting odds ratio (ROR) in Vigibase. Table S3. Clinical characteristics of patients with ICI-associated cerebral vasculitis collected from VigiBase. Table S4. Clinical characteristics of patients with ICI-associated myasthenia gravis collected from VigiBase. Table S5. Clinical characteristics of patients with ICI-associated encephalitis/myelitis collected from VigiBase. Table S6. Clinical characteristics of patients with ICI-associated Guillain-Barre Syndrome collected from VigiBase. Table S7. Clinical characteristics of patients with ICI-associated non-infectious meningitis collected from VigiBase. (DOCX 38 kb) [file 40425_2019_617_MOESM1_ESM.docx]

| Table S1. Neuro-psychiatric adverse events grouping as a function of Medical Dictionary for Regulatory Activities (MedDRA) Classification Version 21.0. | |
| --- | --- |
| Groups | **MedDRA Terms used** |
| Noninfectious meningitis | Meningitis (PT) and/or Meningitis aseptic (PT) |
| Noninfectious encephalitis and/or myelitis | Encephalitis NEC (HLT) and/or Myelitis transverse (PT) and/or Myelitis (PT) |
| Noninfectious encephalopathy/delirium | Noninfectious encephalopathy/delirium (SMQ narrow) |
| Epilepsy/Seizures | Seizures (incl subtypes) (HLGT) |
| Dementia | Dementia (SMQ narrow) |
| Headaches and migraines | Headaches (HLGT) |
| Sleep disturbances | Sleep disturbances (incl subtypes) (HLGT) |
| Coma | Coma states (HLT) |
| Demyelination (excluding Guillain-Barre syndrome) | Demyelinating disorders (HLGT) |
| Spinal cord and nerve root disorders | Cervical spinal cord and nerve root disorders (HLT) and/or lumbar spinal cord and nerve root disorders (HLT) and/or spinal cord and nerve root disorders traumatic (HLT) |
| Increased intracranial pressure and hydrocephalus | Increased intracranial pressure and hydrocephalus (HLGT) |
| Benign nervous system neoplasms | Nervous system neoplasms benign (HLGT) |
| Central nervous system vascular ischemia | Ischaemic central nervous system vascular conditions (SMQ narrow) |
| Central nervous system vascular hemorrhage | Haemorrhagic central nervous system vascular conditions (SMQ narrow) |
| Cerebral artery vasculitis | Vasculitis cerebral (PT) and/or Temporal arteritis (PT) |
| Movement disorders (including parkinsonism and dyskinesia) | Movement disorders (incl parkinsonism) (HLGT) |
| Extrapyramidal syndrome | Extrapyramidal syndrome (SMQ broad) |
| Sensory abnormalities | Paraesthesias and dysaesthesias (HLT) and/or Sensory abnormalities NEC (HLT) |
| Speech and language abnormalities | Speech and language abnormalities (HLT) |
| Vertigos | Vertigos NEC (HLT) |
| Cranial Nerve Disorders | Cranial nerve disorders (excl neoplasms, HLGT) |
| Peripheral neuropathy | Peripheral neuropathy (SMQ narrow) |
| Acute polyneuropathies | Acute polyneuropathies (HLT) |
| Chronic polyneuropathies | Chronic polyneuropathies (HLT) |
| Mononeuropathies | Mononeuropathies (HLT) |
| Guillain-Barre syndrome | Guillain-Barre syndrome (SMQ narrow) |
| Neuromuscular junction dysfunction | Neuromuscular junction dysfunction (HLT) |
| Anticholinergic syndrome | Anticholinergic syndrome (SMQ broad) |
| Psychosis and psychotic disorders | Psychosis and psychotic disorders (SMQ broad) |

*Abbreviations*: HL(G)T: High Level (Group) Term; incl: including; NEC: Not elsewhere classified; PT: Preferred Term; SOC: System Organ Class; SMQ: Standardized MedDRA Query.

***Table S2***: Example of calculating reporting odds ratio (ROR) in Vigibase

|  | Reports with the AE | Reports without the AE |
| --- | --- | --- |
| Reports with suspected class of drugs (e.g. anti-PD-1/PD-L1) | A | B |
| Reports with comparator class of drugs (e.g. anti-CTLA-4) | C | D |

A: Number of reports of drug-induced AE of interest (e.g; myasthenia gravis) associated with the given group of drug (anti-PD-1/PD-L1).

B: Number of reports of all other AE (e.g; all AE excluding myasthenia gravis) associated with the given group of drug (anti-PD-1/PD-L1).

C: Number of reports of AE of interest (e.g; myasthenia gravis) associated with a comparator group of drugs (anti-CTLA-4).

D: Number of reports of other AE (e.g; all AE excluding myasthenia gravis) associated with a comparator group of drugs (anti-CTLA-4).

ROR = (A/C)/(B/D) = AD/BC.

***Table S3****:* Clinical characteristics of patients with ICI-associated **cerebral vasculitis** collected from VigiBase

| **Characteristics** | ***N* (%)** | **Data available,**  ***N* (%)** |
| --- | --- | --- |
| **Reporting region**  Americas  Europe  Asia  Oceana | 17 (50.00)  16 (47.06)  1 (2.94)  0 (0.00) | 34 (100.00) |
| **Reporting year**  2018 (through November)  2017  2016  2015  2014  2012-2013 | 15 (44.12)  7 (20.59)  5 (14.71)  3 (8.82)  0 (0.00)  4 (11.76) | 34 (100.00) |
| **Reporters**  Healthcare professional  Non-healthcare professional | 31 (91.18)  3 (8.82) | 34 (100.00) |
| **Gender**  Male  Female | 15 (46.88)  17 (53.13) | 32 (94.12) |
| **Age at onset,** mean ± SD, years  [min-max] | 68.48±12.50  [36-88] | 25 (73.53) |
| **Drugs**  *Monotherapy with Anti PD-1/PD-L1*   - *Nivolumab* - *Pembrolizumab* - *Atezolizumab* - *Durvalumab* - *Avelumab*   *Monotherapy with Anti CTLA-4*   - *Ipilimumab*   *Combination therapy*   - *Nivolumab + Ipilimumab* - *Pembrolizumab + Ipilimumab* - *Tremelimumab + Durvalumab* | 20 (58.82)  9 (45.00)  9 (45.00)  1 (5.00)  0 (0)  1 (5.00)  10 (29.41)  10 (100)  4 (11.76)  3 (75.00)  1 (25.00)  0 (0) | 34 (100.00) |
| **Suspected Drugs***  Only ICI  ICI + 1 other drug  ICI + ≥2 other drugs | 30 (88.24)  3 (8.82)  1 (2.94) | 34 (100.00) |
| **Number of ICI administration before onset***,* median [IQR],  [min-max] | 4, [2.5-6]  [1-21] | 15 (44.12) |
| **Severe AE** | 31 (100.0) | 31 (91.18) |
| **Indications**  Malignant melanoma  Lung cancer  Non-small cell lung cancer  Small cell lung cancer  Not specified  Colorectal cancer  Merkel cell carcinoma  Renal cell carcinoma  Skin cancer- Unspecified | 11 (44.00)  9 (36.00)  7 (28.00)  0 (0.00)  2 (8.00)  2 (8.00)  1 (4.00)  1 (4.00)  1 (4.00) | 25 (73.53) |
| **Main CNS Toxicity:**  Temporal arteritis  Vasculitis cerebral  Retinal vasculitis | 23 (67.65)  8 (23.53)  3 (8.82) | 34 (100.0) |
| **Time to irAE onset,** days**:**  Median, [IQR]  [min-max]  *Data available* | 80 [47-113]  [14-300] | 15 (44.12) |
| **Death** | 1 (2.94) | 34 (100.0) |
| **Concurrent neurologic symptoms/syndromes**  Myasthenia gravis  Encephalitis/myelitis  Cerebral vasculitis  Guillain Barre syndrome  Peripheral Neuropathy  Meningitis  Demyelination  Seizure  Stroke  Blindness (unilateral or bilateral)  Coma/loss of consciousness | 0 (0)  1 (2.94)  N/A  0 (0)  0 (0)  0 (0)  0 (0)  0 (0)  1 (2.94)  3 (8.82)  2 (5.88) | 34 (100.0) |
| **Other irAEs**  Colitis/diarrhea  Pneumonitis  Myocarditis  Myositis  Dermatitis  Thyroiditis/hypothyroidism  Hypophysitis/hypopituitarism  Hepatitis  Nephritis  Other  **None** | 3 (8.82)  1 (2.94)  0 (0)  0 (0)  0 (0)  1 (2.94)  0 (0)  0 (0)  0 (0)  2 (5.88)  28 (82.35) | 34 (100.0) |

* Other concomitant reported suspected medications were Bevacizumab (n:3), Brentuximab vedotin (n:2), Carboplatin (n:2), Cobimetinib (n:2), Aldesleukin (n:1), Azithromycin (n:1), Cabozantinib (n:1), Crizotinib (n:1), Entinostat (n:1), Etanercept (n:1), Ethanol (n:1), Etoposide (n:1), Golimumab (n:1), Influenza vaccine (n:1), Interferon alfa-2b (n:1), Metronidazole(n:1), Oxycodone (n:1), Pemetrexed, (n:1), Radium (n:1), Simvastatin (n:1), Tizanidine (n:1), and Drug names under assessment (n:4).

***Table S4****:* Clinical characteristics of patients with ICI-associated **myasthenia gravis** collected from VigiBase

| **Characteristics** | ***N* (%)** | **Data Available**  ***N* (%)** |
| --- | --- | --- |
| **Reporting Region**  North America  Europe  Australia  Asia  Africa | 103 (45.18)  64 (28.07)  12 (5.26)  48 (21.05)  1 (0.44) | 228 (100.00) |
| **Reporters**  Healthcare Professional  Non-Healthcare Professional | 188 (87.44)  27 (12.56) | 215 (94.30) |
| **Suspected Drugs**^1^  Only ICI  ICI + 1 other drug  ICI + 2+ drugs | 216 (94.74)  10 (4.39)  2 (0.88) | 228 (100.00) |
| **Number of ICI doses before onset**, median [IQR]  [min – max] | 2 [2 – 3]  [1 – 21] | 19 (8.33) |
| **Severe AE** | 215 (100.00) | 215 (94.30) |
| **Indication for ICI**  Melanoma  Lung  Non-small cell lung cancer  Small cell lung cancer  Not specified  Renal  Bladder  Gastric  Head and neck squamous cell  Thymic  Adenocarcinoma, unknown primary  Hematologic cancers  Colorectal  Merkel  Liver  Bone  Thyroid  **Prostate**  **Uterine** | 47 (25.54)  73 (39.67)  48 (26.09)  6 (3.26)  19 (10.33)  25 (13.59)  9 (4.89)  6 (3.26)  6 (3.26)  4 (2.17)  3 (1.63)  2 (1.09)  2 (1.09)  2 (1.09)  1 (0.54)  1 (0.54)  1 (0.54)  1 (0.54)  1 (0.54) | 184 (80.70) |
| **Main CNS Toxicity**  Myasthenia Gravis  Ocular Myasthenia  Myasthenia Gravis Crisis  Myasthenic Syndrome  Myasthenia Gravis + Myasthenic Syndrome  Myasthenia Gravis + Myasthenia Gravis Crisis | 164 (71.93)  45 (19.74)  11 (4.82)  5 (2.19)  2 (0.88)  1 (0.44) | 228 (100.00) |

^1^ Other concomitant reported suspected medications were Ambenonium, Axitinib, Azathioprine, Bevacizumab, Carboplatin, Cefoperazone;Sulbactam, Entinostat, Gemcitabine, Immunoglobulin human normal, Leuprorelin, Paracetamol, Peginterferon alfa-2a, Prednisolone, Pyridostigmine, Thiamazole, and Thrombomodulin alfa (n = 1 for all).

Abbreviations: CTLA-4, cytotoxic T-lymphocyte-associated protein 4; ICI, immune checkpoint inhibitor; IQR, interquartile range; irAE, immune related adverse event; [min-max], minimum-maximum; PD-1, programmed cell death protein 1; PD-L1, programmed cell death ligand 1; SD, standard deviation

***Table S5****:* Clinical characteristics of patients with ICI-associated **encephalitis/myelitis** collected from VigiBase

| **Characteristics** | ***N* (%)** | **Data available,**  ***N* (%)** |
| --- | --- | --- |
| **Reporting region**  Americas  Europe  Asia  Oceana | 129 (51.60)  92 (36.80)  20 (8.00)  9 (3.60) | 250 (100.00) |
| **Reporters**  Healthcare professional  Non-healthcare professional | 209 (87.08)  31 (12.92) | 240 (96.00) |
| **Suspected Drugs***  Only ICI  ICI + 1 other drug  ICI + ≥2 other drugs | 230 (92.00)  16 (6.40)  4 (1.60) | 250 (100.00) |
| **Number of ICI administration before onset***,* median [IQR],  [min-max] | 3, [1-8.5]  [1-34] | 51 (20.40) |
| **Severe AE** | 240 (100.0) | 240 (96.00) |
| **Death** | 33 (13.2) | 250 (100.00) |
| **Indications**  Lung cancer  Non-small cell lung cancer  Small cell lung cancer  Not specified  Malignant melanoma  Hematologic cancer and lymphoma  Renal cell carcinoma  Malignant neoplasm non-specified  Urothelial cancer  Gastrointestinal cancer  Breast cancer  Bile duct cancers and Cholangiocarcinoma  Hepatocellular carcinoma  Cervix carcinoma  Mesothelioma  Squamous cell carcinoma of head and neck | 91 (39.39)  53 (22.94)  5 (2.16)  33 (14.29)  54 (23.38)  19 (8.23)  14 (6.06)  12 (5.19)  7 (3.03)  4 (1.73)  2 (0.87)  2 (0.87)  2 (0.87)  1 (0.43)  1 (0.43)  1 (0.43) | 231 (85.20) |
| **Main CNS Toxicity:**  Encephalitis  Myelitis  Paraneoplastic Encephalomyelitis  CNS Inflammation | 225 (90.00)  20 (8.00)  4 (1.60)  1 (0.40) | 250 (100.0) |

* Other concomitant reported suspected medications were Bevacizumab (n:3), Brentuximab vedotin (n:2), Carboplatin (n:2), Cobimetinib (n:2), Aldesleukin (n:1), Azithromycin (n:1), Cabozantinib (n:1), Crizotinib (n:1), Entinostat (n:1), Etanercept (n:1), Ethanol (n:1), Etoposide (n:1), Golimumab (n:1), Influenza vaccine (n:1), Interferon alfa-2b (n:1), Metronidazole(n:1), Oxycodone (n:1), Pemetrexed, (n:1), Radium (n:1), Simvastatin (n:1), Tizanidine (n:1), and Drug names under assessment for who-dd (n:4).

***Table S6****:* Clinical characteristics of patients with ICI-associated **Guillain-Barre Syndrome** collected from VigiBase

| **Characteristics** | ***N* (%)** | **Data Available, *N* (%)** |
| --- | --- | --- |
| **Reporting Region** North America Europe Oceana Asia | 65 (53.3) 42 (34.4)  10 (8.2)  5 (4.1) | 122 (100) |
| **Reporters** Healthcare personnel Non-healthcare personnel | 92 (80) 23 (20) | 115 (94) |
| **Suspected Drugs*** Only ICI ICI + 1 other drug ICI + >2 other drugs | 114 (93.4) 5 (4.1) 3 (2.5) | 122 (100) |
| **Number ICI admin before onset** Median [min – max] | 3 [1-31] | 21 (17.2) |
| **Indications** Malignant Melanoma Lung Cancer  Non-small cell lung cancer  Small cell lung cancer  Not specified Renal Cell Carcinoma Urothelial Cancer Hematologic cancer and lymphoma Uterine cancer Head/neck cancer Mesothelioma Simultaneous Lung (unspecified) and Melanoma | 65 (67.7)  18 (18.75)  12 (12.5)  0  6 (6.25) 5 (5.2) 3 (3.1) 1 (1.0) 1 (1.0) 1 (1.0) 1 (1.0) 1 (1.0) | 96 (78.7) |
| **Main CNS Toxicity:**  Guillain-Barre Syndrome  Demyelinating Polyneuropathy  Chronic Inflammatory Demyelinating Polyneuropathy  Acute Axonal Neuropathy  Miller Fisher Syndrome | 97 (79.51)  15 (12.30)  6 (4.92)  3 (2.46)  1 (0.82) | 122 (100.00) |

*Other concomitant reported suspected medications were: dabrafenib(4), trametinib(3), vemurafenib(2), influenza vaccine(1), interleukins(1), cyclophosphamide(1), fludarabine(1), and brentuximab(1).

***Table S7****:* Clinical characteristics of patients with ICI-associated **non-infectious meningitis** collected from VigiBase

| **Characteristics** | ***N* (%)** | **Data Available** |
| --- | --- | --- |
| **Reporting Region** North America Europe Oceana Asia | 36 (50.00) 29 (40.28) 1 (1.39) 6 (8.33) | 72 (100) |
| **Reporters** Healthcare Personnel Non-healthcare Personnel | 63 (90.00) 7 (10.00) | 70 (97.2) |
| **Suspected Drugs** Only ICI ICI + 1 other drug | 65 (90.27) 7 (9.72) | 72 (100) |
| **Number of ICI admin before onset**  **Median [IQR]**  **[min-max]** | 1 [1 -4.5]  [1-20] | 17 (23.61) |
| **Time to onset, weeks** Median [min, max] | 10 [0 - 49] | 23 (31.94) |
| **Indication** Malignant Melanoma Lung Cancer  Non-small cell lung cancer  Small cell lung cancer  Not specified Renal Cell Cancer Hodgkin's Disease Bladder Carcinoma Bile Duct Cancer SCC of head/neck Glioblastoma B-cell lymphoma Adenocarcinoma (unspecified) Breast Cancer Concurrent Lung (unspecified) and Bladder Cancer | 38 (60.12) 7 (11.11)  5 (7.94)  0 (0.00)  2 (3.17)  5 (7.94) 3 (4.76) 3 (4.76) 1 (1.59) 1 (1.59) 1 (1.59) 1 (1.59) 1 (1.59) 1 (1.59) 1 (1.59) | 63 (87.5) |
| **Main CNS Toxicity:**  Aseptic Meningitis  Meningitis | 40 (55.56)  32 (44.44) | 72 (100.00) |

*Other concomitant reported suspected medications were cabozantinib(n:1), cobimetinib(n:1), dacarbazine(n:1), dendritic cells/cytokine induced killer cells(n:1), rituximab (n:1) and an unspecified investigational drug (n:2).
